# Associations between social media addiction, psychological distress, and food addiction among Taiwanese university students

**DOI:** 10.1186/s40337-023-00769-0

**Published:** 2023-03-21

**Authors:** Po-Ching Huang, Janet D. Latner, Kerry S. O’Brien, Yen-Ling Chang, Ching-Hsia Hung, Jung-Sheng Chen, Kuo-Hsin Lee, Chung-Ying Lin

**Affiliations:** 1 Institute of Allied Health Sciences, College of Medicine, National Cheng Kung University, No. 1, University Rd., East Dist., Tainan, 701401 Taiwan; 2 Department of Psychology, University of Hawaii at Manoa, 2500 Campus Road, Honolulu, HI 96822 USA; 3 School of Social Sciences, Monash University, 20 Chancellors Walk, Clayton, VIC 3800 Australia; 4 Department of Family Medicine, Cardinal Tien Hospital, No. 362, Zhongzheng Rd., Xindian Dist., New Taipei, 231009 Taiwan; 5 Department of Physical Therapy, College of Medicine, National Cheng Kung University, No. 1, University Rd., East Dist., 701401 Tainan, Taiwan; 6 Department of Medical Research, E-Da Hospital, I-Shou University, No.1, Yida Rd, Yanchao District, 824005 Kaohsiung, Taiwan; 7 Department of Emergency Medicine, E-Da Hospital, I-Shou University, No.1, Yida Rd., Yanchao Dist., 840301 Kaohsiung, Taiwan; 8 School of Medicine, I-Shou University, No.8, Yida Rd., Yanchao Dist., 824005 Kaohsiung, Taiwan; 9 Biostatistics Consulting Center, National Cheng Kung University Hospital, College of Medicine, National Cheng Kung University, No. 1, University Rd., East Dist., Tainan, 701401 Taiwan; 10 Department of Public Health, National Cheng Kung University Hospital, College of Medicine, National Cheng Kung University, No. 1, University Rd., East Dist., Tainan, 701401 Taiwan; 11 Department of Occupational Therapy, College of Medicine, National Cheng Kung University, No. 1, University Rd., East Dist., Tainan, 701401 Taiwan

**Keywords:** Social media addiction, Food addiction, Mediation model, Psychological distress, Taiwan

## Abstract

**Background:**

Worldwide, 60% of people use social media. Excessive and/or addictive use of social media termed “problematic social media use”, has been reported to negatively influence psychological and physiological health. Therefore, we proposed an illustrated model to investigate the associations between social media addiction, psychological distress and food addiction among Taiwanese university students.

**Methods:**

A total of 598 participants (mean age = 22.8 years) completed an online survey comprising the Bergen Social Media Addiction Scale (BSMAS) assessing social media addiction, the Depression Anxiety and Stress Scale (DASS-21) assessing psychological distress, and the Yale Food Addiction Scale 2.0 (YFAS 2.0) assessing food addiction.

**Results:**

Structural equation modeling showed the significant associations between BSMAS and DASS-21 (standardized coefficient [β] = 0.45; *p* < 0.01) and between DASS-21 and YFAS 2.0 (β = 0.43; *p* < 0.01). In addition, mediation effect with 100 bootstrapping samples showed the indirect effect of DASS-21 in the association between BSMAS and YFAS 2.0

**Conclusions:**

The present study details the relationships between social media addiction and psychological distress as well as food addiction. The results suggest the need for interventions aimed at reducing these negative outcomes. Coping strategies for improving self-control or reducing weight-related stigma, such as food consumption monitoring or mindfulness, could be adopted for at-risk individuals to address these problems.

## Abstract (summary)

Social media addiction has been found to have psychological and physiological impacts on individuals’ health. In order to better understand the role of social media addiction, the present study constructed a model to investigate the potential mechanism of social media addiction in affecting the individuals’ food addiction level. The findings showed a clear pathway between social media addiction and food addiction with the involvement of psychological distress. Accordingly, we suggested that individuals with the potential risk of social media addiction should pay attention to their psychological status and food intake. The potential effect of weight-related stigmatization would also need to be considered, strategies such as mindfulness or food consumption monitoring would be beneficial to address the issues.

## Introduction

With the development of information technology, Internet-based social media networking has rapidly increased [1]. A recent report indicated that 59.3% of the total global population or approximately 4.7 billion people were social media users in 2022 [2]. Both positive and negative effects of social media use have been found among social media users. Positive effects such as joy and relaxation [3], information exchange [4], and increased physical activity [5] were found in some groups of social media users. However, negative social media effects such as psychological distress [3], excessive use [6], and sedentary lifestyle [7] were also reported in small groups of individuals leading to poorer mental health [8]. The group of individuals experiencing negative outcomes from social media use is considered to have a specific type of problematic Internet use (i.e., problematic social media use, [PSMU]). The present study sought to build on several previous studies in the field [9–11] by focusing on social media use as individuals’ recreational social interaction via online platforms, such as web sites or smartphone applications that contain user-generated content. The rapidly increasing number of social media users [2] has made PSMU a global issue that cannot be ignored.

PSMU is defined as the excessive interaction and networking on social media networking, to the extent that these behaviors impair other important activities such as education, work, interpersonal relationship, and/or psychological health and well-being [6, 12–14]. Prior cross-sectional research has documented that PSMU was associated with poor sleep quality [6, 15], psychological function [6, 16, 17], as well as other addictive behaviors [14, 17]. Furthermore, some longitudinal research showed a prolonged influence of PSMU on increasing sleep disturbance, depression and anxiety [18, 19], even suicidal-related outcomes [20]. Among these negative influences, the association between PSMU and psychological distress (e.g., depression or anxiety) is well-reported [6, 16–19]. One study reported that psychological distress derived from PSMU is more likely to be developed via internal (e.g., loneliness) rather than external process (e.g., social isolation) [14]. More specifically, individuals’ self-regulation and use-expectation were reported to mediate the significant association between PSMU and depressive symptoms [21]. In addition, individuals with PSMU reported sleep interruption [6, 15, 19] and lower physical activity level [22], which may further worsen their mental health status [6, 15]. They also tend to engage in excessive self-comparison and prejudiced self-image [23], as a result of continually comparing themselves to the idealized portraits posted on social media [14, 23]. These physical and psychological impacts may exacerbate vulnerability and increase the risk of developing psychological distress [14].

Psychological health is one of the major health issues which may impair individuals’ functional performance [24]. Individuals with mental health disturbances may be impaired in employment, educational, or relationship domains [24, 25], subsequently exacerbating their perceived stress and risk of burnout [25]. For those with relatively poor emotional regulation, instinctive avoidance [26] of this unpleasant feeling may act as a coping strategy [24] and prompt the development of addictive behaviors such as problematic use of the Internet [27] or food addiction [28, 29]. Studies have reported a robust association between mental health symptoms and food addiction [28, 29]. Food addiction refers to individuals’ uncontrollable desire to obtain food [29]. Specifically, as acts of comfort-seeking behavior, emotional eating and other disordered eating behaviors are commonly observed as a stress-alleviating strategy [30, 31]. Psychological distress (e.g., depression or anxiety) is highly correlated to the development of food addiction [29, 31] due to increased vulnerability [29].

**Fig. 1:**
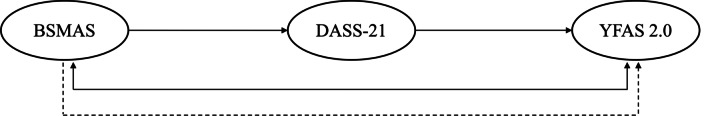
Proposed model to illustrate the potential mechanism and the mediation effect of social media addiction affecting food addiction. Solid line indicates direct effect; dash line indicates indirect effect. BSMAS = Bergen Social Media Addiction Scale; DASS-21 = Depression Anxiety and Stress Scale; YFAS 2.0 = Yale Food Addiction Scale 2.0

There are more than 21.4 million (equal to 89.4% of the total population) social media users [32] in Taiwan in 2022, which is higher than global statistics (59.3%) in 2022 [2]. Additionally, when compared to other age groups, young adults, especially those of university age [33], are much likelier to develop problematic Internet use, because university students may be living independently for the first time [34]. Without monitoring and supervision by parents [34], along with peer pressure to use technology and engage in social comparison behavior (i.e., comparing ourselves to others) [35], university students may lose their boundaries or restrictions and become more vulnerable to developing addictive use (e.g., of the Internet or food intake) than older or younger peers [33]. However, to the present authors’ knowledge, currently there is no study investigating the potential association between social media addiction, psychological distress and food addiction. There is also a lack of research investigating the connections between problematic social media use and food addiction level among the Taiwanese population. Therefore, we proposed a model (Fig. 1) that aimed to investigate the relationship between social media addiction, psychological distress, and food addiction. Moreover, mediation effects of psychological distress were examined when the aforementioned relationships were tested. Accordingly, we hypothesized that (1) social media addiction is positively correlated to psychological distress; (2) psychological distress is positively associated with food addiction; (3) psychological distress mediates the relationship between social media addiction and food addiction.

## Method

### Participants

Participants who met the following inclusion criteria were recruited into the present study: (i) being 20 years old or above; (ii) registered in the program (either undergraduate or postgraduate regardless of study major) of any university in Taiwan when they completed the survey; (iii) had at least one active social media account (e.g., Facebook or Instagram); and (iv) able to read Chinese. The majority of participants were male students (65.38%) with a relatively young age (mean age = 22.8 years; SD = 3.75; ranged from 20 to 45) and an average body mass index (BMI) of 21.98 kg/m^2^ (SD = 3.71). Specifically, 87 participants (14.5%) were defined as low weight (BMI lower than 18.5); 393 participants (65.7%) were defined as average weight (BMI ranged from 18.5 to 24.9); 118 participants (19.7%) were defined as high weight (BMI higher than 25.0) [36].

### Measures

#### Demographics and social media usage

Demographics information was collected, including age, gender, along with weekly time spent on social media. Moreover, self-reported height and weight were used to calculate BMI (kg/m^2^).

#### Social media addiction

The Bergen Social Media Addiction Scale (BSMAS) [37] was used to assess social media addiction. The BSMAS contains 6 items with a 1 to 5 Likert-like scale (1 = seldom; 5 = very often). The item scores were summed to generate a total BSMAS score ranged from 5 to 30, with higher scores indicating more severe social media addiction. A sample item is “*I feel an urge to use social media more and more*”. The psychometric properties (including construct validity, concurrent validity, test–retest reliability, and internal consistency) of the Chinese version of the BSMAS have been found satisfactory in prior research [38] and demonstrated an excellent internal consistency in the present study (Cronbach’s alpha [α] = 0.96).

#### Psychological distress

The Depression Anxiety and Stress Scale (DASS-21) [39] was used to assess psychological distress (i.e., depression, anxiety and stress). The DASS-21 contains 21 items (seven items for each type of distress) with a 0 to 3 Likert-like scale (0 = never; 3 = almost always). In the present study, the overall psychological distress score was used. Therefore, the 21 item scores were summed and multiplied by 2 to generate a total score ranged from 0 to 126 [40]. A higher score indicates more severe psychological distress. A sample item is “*I found it difficult to relax*”. The psychometric properties (including construct validity, concurrent validity, test–retest reliability, and internal consistency) of the Chinese version of DASS-21 have been found satisfactory in prior research [41] and demonstrated an excellent internal consistency in the present study (α = 0.98).

#### Food addiction

The Yale Food Addiction Scale 2.0 (YFAS 2.0) [42] was used to assess food addiction. The YFAS 2.0 contains 35 items with a 0 to 7 Likert-like scale (0 = never; 7 = everyday). YFAS 2.0 adopted an unique scoring method [42] with the 35 items converted into 11 symptoms with a 0–1 dichotomous scale (0 indicates *non-endorsed*; 1 indicates *endorsed*) to obtain a total scores ranging from 0 to 11. A higher score suggests a more severe level of food addiction. A sample item is “*I have problems with my family and friends because of how much I ate*”. The psychometric properties (including construct validity, concurrent validity, test–retest reliability, and internal consistency) of the Chinese version of the YFAS 2.0 have been found satisfactory in prior research [43], with good internal consistency in the present study (α = 0.87).

### Procedure

An online survey hosted on *Google Forms* was distributed using snowball sampling from August to September, 2021. More specifically, the survey link was sent to various university departments and those who received the survey link were encouraged to disseminate the survey information via the weblink or QR code. The consent form was shown on the first page of the online survey. By clicking the “*agree*” icon indicated the participants gave their informed consent to participate in the present study. In addition, the participants received 100 New Taiwan dollars reimbursement (approximately $3.3 US) after they completed all the survey questions. The present study was approved by the Institutional Review Board in the Chi Mei Medical Center (IRB Serial No.: 11007-006) and the Human Research Ethics Committee in the National Cheng Kung University (Approval No.: NCKU 144 HREC-E-109-551-2).

### Statistical analysis

Demographics and the scores on the three measures were first summarized using descriptive analysis. Then, the correlation coefficients between variables were computed using Pearson’s correlation. Structural equation modeling (SEM) with the estimator of diagonally weighted least squares was used to examine whether the data fit with the proposed models. Because the proposed models contained mediation effect, 100 bootstrapping samples were set to examine the mediation effect. Fit indices of comparative fit index (CFI), Tucker–Lewis index (TLI), root mean square error of approximation (RMSEA) and standardized root mean squared residual (SRMR) were used to verify if the model was supported. The levels of both CFI and TLI should be > 0.95 and those of RMSEA and SRMR should be < 0.08 [44]. In addition, a total of 2 competing models considering the present of different direct effects were illustrated for model comparisons. The model with lower expected cross validation index (ECVI) is suggested to be the best fitting model. A conceptual model for SEM testing is illustrated in Fig. 1 and the following describes the structures of the 2 competing models. Model 1: Model without correlation between social media addiction and food addiction; Model 2: Based on Model 1 with one additional correlation between social media addiction and food addiction (this is also the proposed model with all the hypotheses mentioned in the Introduction). The SEM was performed using the *lavaan* package in the R software [45] and the remaining data analyses were performed using the SPSS 22.0 (IBM, Corp., NY: Armonk).

## Results

Table 1 displays the background of participants (n = 598) and the mean scores of measures. The present sample reported an average 3.21 h of daily social media use. Moreover, the mean scores of the measures were 15.90 (SD = 4.78) out of 30 for BSMAS, 26.94 (SD = 25.39) out of 126 for DASS-21, and 1.92 (SD = 2.99) out of 11 for YFAS 2.0. Table 2 displays the correlations between the studies variables. In sum, all three measures were significantly correlated to BMI (*r* = 0.10–0.22) and each other (*r* = 0.44–0.54). In addition, BSMAS was significantly correlated with time spent on social media (*r* = 0.29, *p*-values < 0.01).

**Table 1:** Participants’ characteristics (N = 598)

	Mean (SD) or n (%)
Age (year)	22.8 (3.75)
Gender (male)	391 (65.38)
Body mass index (kg/m^2^)	21.98 (3.71)
Time spend on social media (hrs/day)	3.21 (2.44)
BSMAS score (possible score range: 5–30)	15.90 (4.78)
DASS-21 score (possible score range: 0–126)	26.94 (25.32)
YFAS 2.0 score (possible score range: 0–11)	1.92 (2.99)

*BSMAS* Bergen Social Media Addiction Scale, *DASS-21* Depression Anxiety and Stress Scale, *YFAS 2.0* Yale Food Addiction Scale 2.0

**Table 2:** Correlations between study variables (N = 598)

		1	2	3	4	5	6	7
1	Age	–						
2	Gender	− 0.02 (0.72)	–					
3	BMI	**0.10 (0.01)**	**0.26 (< 0.01)**	–				
4	Time spend on social media	− 0.04 (0.30)	− 0.06 (0.28)	0.04 (0.30)	–			
5	BSMAS	0.000 (0.99)	− 0.01 (0.78)	**0.10 (0.02)**	**0.29 (< 0.01)**	–		
6	DASS-21	0.01 (0.77)	0.01 (0.83)	**0.13 (< 0.01)**	0.03 (0.46)	**0.44 (< 0.01)**	–	
7	YFAS 2.0	0.01 (0.75)	0.04 (0.35)	**0.22 (< 0.01)**	0.07 (0.08)	**0.45 (< 0.01)**	**0.54 (< 0.01)**	–

*BSMAS* Bergen Social Media Addiction Scale, *DASS-21* Depression Anxiety and Stress Scale, *YFAS 2.0* Yale Food Addiction Scale 2.0. Significant correlations are shown in **bold**

**Fig. 2:**
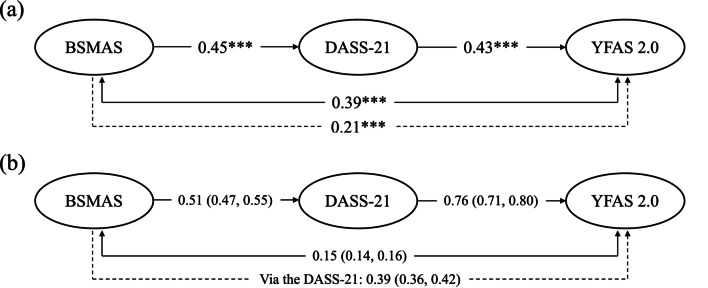
Mediation effect with 100 bootstrapping samples of investigated variables. Age and gender were controlled in the model (N = 598). Solid line indicates direct effect; dash line indicates indirect effect. **a** Coefficients reported using standardized coefficient. *p < 0.05; **p < 0.01; ***p < 0.001; **b** coefficients reported using unstandardized coefficients with 95% confidence interval in parentheses. BSMAS = Bergen Social Media Addiction Scale; DASS-21 = Depression Anxiety and Stress Scale; YFAS 2.0 = Yale Food Addiction Scale 2.0

Table 3 demonstrates the fit indices between the two competing SEM models. Briefly, Model 2 demonstrated a relatively good fit when compared to all the other models, with the support of all fit indices (CFI = 0.995; TLI = 0.995; RMSEA = 0.02; SRMR = 0.06; and ECVI = 4.85). The SEM results, shown in Fig. 2, demonstrated the significant correlations of BSMAS to DASS-21 (standardized coefficient [β] = 0.48; *p* < 0.01) and DASS-21 to YFAS 2.0 (β = 0.43; *p* < 0.01). In addition, BSMAS has an additionally significant association with YFAS 2.0 (β = 0.39; *p* < 0.01). Moreover, the mediation effect of DASS-21 (β = 0.21; *p* < 0.01) was found in explaining the association between BSMAS and YFAS 2.0 when age, gender and BMI were controlled. More specifically, the unstandardized coefficient (95% bootstrapping CI) was 0.15 (0.14, 0.16) for indirect effect between BSMAS and YFAS 2.0 via DASS-21. Lastly, BSMAS indirectly mediated the YFAS 2.0 via the DASS-21 with the unstandardized coefficient (95% bootstrapping CI) of 0.39 (0.36, 0.42).

**Table 3:** Fit indices of two competing models

	Model 1	Model 2
χ^2^ (df)	3526.73 (2010)	2622.16 (2009)
p-value	< .01	< .01
CFI	0.988	0.995
TLI	0.987	0.995
RMSEA	0.04	0.02
SRMR	0.07	0.06
ECVI	6.36	4.85

Model 1: Proposed model without the correlation between social media addiction and food addiction

Model 2: Proposed model

*CFI* comparative fit index, *TLI* Tucker–Lewis index, *RMSEA* root mean square error of approximation, *SRMR* standardized root mean square residual, *ECVI* expected cross validation index

## Discussion

The present study investigated the potential mechanism by which social media addiction is associated with food addiction among Taiwan university students, and the findings supported all hypotheses. The results showed the significant associations between social media addiction, psychological distress and food addiction, in which mediation effects of psychological distress was significant. Among the two competing models, Model 2 outperformed Model 1. Model 2 performed better because it contains all the hypotheses and an additional correlation between social media addiction and food addiction. Therefore, the correlation between social media addiction and food addiction could be mediated via psychological distress.

The level of social media addiction was significantly associated with individuals’ psychological distress in the present study. Previous studies reported that among different types of problematic use of the Internet, PSMU has the most salient effect on dysregulating emotional management [46, 47]. More specifically, individuals with poor psychological well-being tend to adopt social media use as a coping strategy [48] and may feel compelled to frequent use in order to avoid their problems [49]. The situation becomes a vicious circle, because the behavior of using social media as a form of avoidance may increase vulnerability of individuals to developing PSMU [48]. In turn, the addictive behavior continues to degrade their psychological health [18, 19], which results in a higher level of addictive behavior [49]. This pattern was supported by the present finding. Other studies additionally suggested a causal link between PSMU and depressive symptoms [50] and demonstrated that it is “the number of used social media platforms” rather than “the total used time”, that is associated with psychological symptoms such as depression, anxiety [51], and body image [52]. The results suggest that more detailed consideration is needed for the investigation of potential social media factors that promote psychological distress [51].

The significant association between psychological distress and food addiction found in the present study corroborate previous findings [28, 29, 31, 53, 54]. Two issues may contribute to this association: (1) lack of control over eating and (2) weight stigma. Self-control skills could be a potential explanation for the association [53] because lack of self-control can result in impulsivity and contribute to food addiction [54–56]. Psychological distress (e.g., resulting from receiving negative information from social media) may trigger excessive emotional eating to cope with stress, which could result in food addiction, particularly in the presence of poor self-control skills [57–59].

Another important factor explaining our finding is weight-related stigma. The correlation between weight-related stigma and food addiction had been well-reported [60]. For example, the fear of being stigmatized predicted greater food addiction [61]. Additionally, weight stigmatization may cause psychological distress [62]. Furthermore, the internalization of weight-related stigma and psychological distress may exacerbate the relationship between weight stigma and disordered eating [63]. Social media addiction may increase body dissatisfaction and lead to the internalization of weight-related stigma, which may result in the psychological distress and further amplify food addiction. Indeed, our findings showed that social media addiction was associated with psychological distress and food addiction. Therefore, interventions such as psychological acceptance or mindfulness therapy may reduce the negative impact of weight stigmatization [62].

The present study demonstrated a possible mechanism of PSMU in relation to food addiction among university students, which included the involvement of psychological distress. However, the present study had several limitations. First, the self-reported variables may be subject to social desirability bias (e.g., participants might have underestimated their social media usage) or recall bias (e.g., participants might not accurately remember their height and weight). Second, the unique characteristics of this university sample may limit generalizability to populations with different age. Third, the cross-sectional design of the present study cannot test the causal directionality of this mechanism and a longitudinal study is merited to provide more information. Despite that, the present findings still provided evidence of the possible negative correlates of social media addiction. Strategies aimed at promoting emotional regulation, self-control skills or reducing the weight stigma, such as mindfulness exercises [53, 62], cognitive restructuring [64], or food consumption monitoring [65], can be taught to the individuals with social media addiction, to lower these unwanted consequential effects.

## Conclusions

The present study investigated the potential mechanism of social media addiction in relation to food addiction among Taiwanese university students. The results showed an association between social media addiction and psychological distress, with psychological distress linked to food addiction. The present study suggests several important negative correlates of social media addiction. Interventions to reduce social media addiction and food addiction might alleviate the negative consequences of these behaviors. Strategies to improve self-control skills or reduce weight stigmatization, such as food-intake monitoring, mindfulness and cognitive restructuring, can be taught to individuals with food addiction to neutralize its negative consequences.

## Data Availability

The data and code that support the present findings are available from the corresponding author upon reasonable request.

## Abbreviations

PSMU: Problematic social media use
BMI: Body mass index
BSMAS: Bergen social media addiction scale
DASS-21: Depression anxiety and stress scale
YFAS 2.0: Yale food addiction scale 2.0
SEM: Structural equation modeling
CFI: Comparative fit index
TLI: Tucker–Lewis index
RMSEA: Root mean square error of approximation
SRMR: Standardized root mean square residual
ECVI: Expected cross validation index
